# An Interplay between Epigenetics and Translation in Oocyte Maturation and Embryo Development: Assisted Reproduction Perspective

**DOI:** 10.3390/biomedicines10071689

**Published:** 2022-07-13

**Authors:** Michal Dvoran, Lucie Nemcova, Jaroslav Kalous

**Affiliations:** Institute of Animal Physiology and Genetics, Czech Academy of Sciences, 277 21 Libechov, Czech Republic; dvoran@iapg.cas.cz (M.D.); nemcova@iapg.cas.cz (L.N.)

**Keywords:** epigenetics, protein translation, oocyte maturation, embryo development

## Abstract

Germ cell quality is a key prerequisite for successful fertilization and early embryo development. The quality is determined by the fine regulation of transcriptomic and proteomic profiles, which are prone to alteration by assisted reproduction technology (ART)-introduced in vitro methods. Gaining evidence shows the ART can influence preset epigenetic modifications within cultured oocytes or early embryos and affect their developmental competency. The aim of this review is to describe ART-determined epigenetic changes related to the oogenesis, early embryogenesis, and further in utero development. We confront the latest epigenetic, related epitranscriptomic, and translational regulation findings with the processes of meiotic maturation, fertilization, and early embryogenesis that impact the developmental competency and embryo quality. Post-ART embryo transfer, in utero implantation, and development (placentation, fetal development) are influenced by environmental and lifestyle factors. The review is emphasizing their epigenetic and ART contribution to fetal development. An epigenetic parallel among mouse, porcine, and bovine animal models and human ART is drawn to illustrate possible future mechanisms of infertility management as well as increase the awareness of the underlying mechanisms governing oocyte and embryo developmental complexity under ART conditions.

## 1. Introduction

The purpose of living organisms’ reproduction is to give rise to a new generation. According to the World Fertility Report from 2015, human fertility has halved in the past 65 years. In the 1950s, the norm was for one woman to give birth to 5 children, whereas in 2015 the average was only 2.5. If this pattern, along with demographic changes, continues, we would gradually face the need to support human fertility clinically by preserving the quality of germ cells, particularly oocytes.

**Figure 1:**
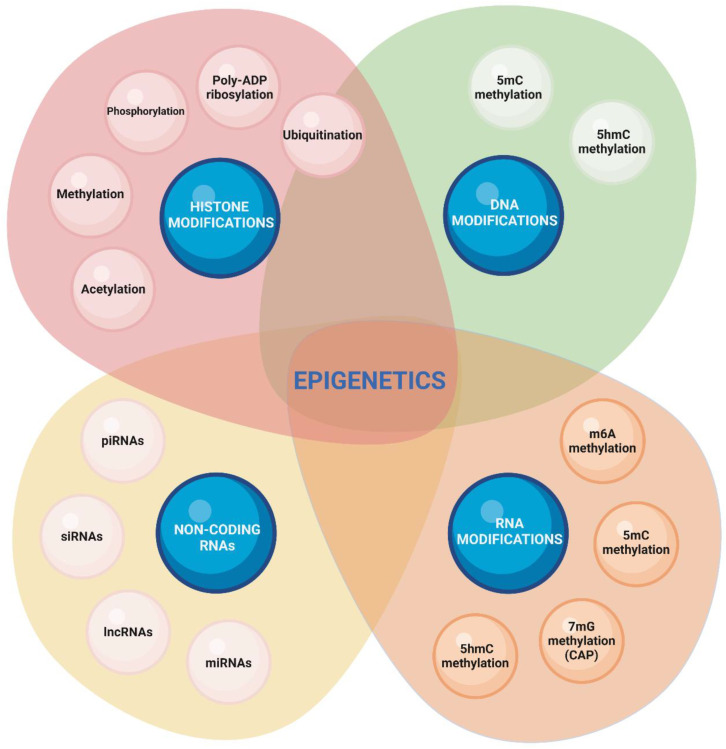
Epigenetic modifications: This diagram shows the complexity of epigenetic processes, which are divided into the following subgroups: DNA modifications—5-methylcytosine (5mC) DNA methylation, 5-hydroxymethylcytosine (5hmC) DNA methylation; Histone modifications—Acetylation, Methylation, Phosphorylation, Poly-ADP ribosylation, Ubiquitination; Non-coding RNA interactions—piwi RNA (piRNA), small interfering RNA (siRNA), long non-coding RNA (lncRNA), micro RNA (miRNA); RNA modifications—6-methyladenosine (6mA) RNA methylation, 5-methylcytosine (5mC) RNA methylation, 7-methylguanosine (7mG) RNA methylation, mRNA CAP, 5-hydroxymethylcytosine (5hmC) RNA methylation. The image was created with BioRender.com.

Female fertility is influenced by genetic background, environmental factors, lifestyle, nutrition, psychosocial setting, and many other factors. The proper understanding of the molecular mechanisms underlying oocyte development is crucial for advancement in assisted reproduction technology. This review is focused on how oocyte and embryo development is affected by epigenetic changes, initially those originating from the in vitro methods of assisted reproduction technology (ART) followed by in utero changes caused by maternal diet, lifestyle, and environment. The epigenome is a complex of chemical compounds that modify or mark a genome but are not part of the DNA itself (Figure 1).

Epigenetic changes may cause temporary or heritable alterations of gene expression. Modifications to the epigenome are reversible and alter gene expression in different ways but do not interfere directly with the DNA genetic code. Epigenetic changes are categorized into DNA, RNA, histone modifications, and changes controlled by non-coding RNAs.

**Figure 2:**
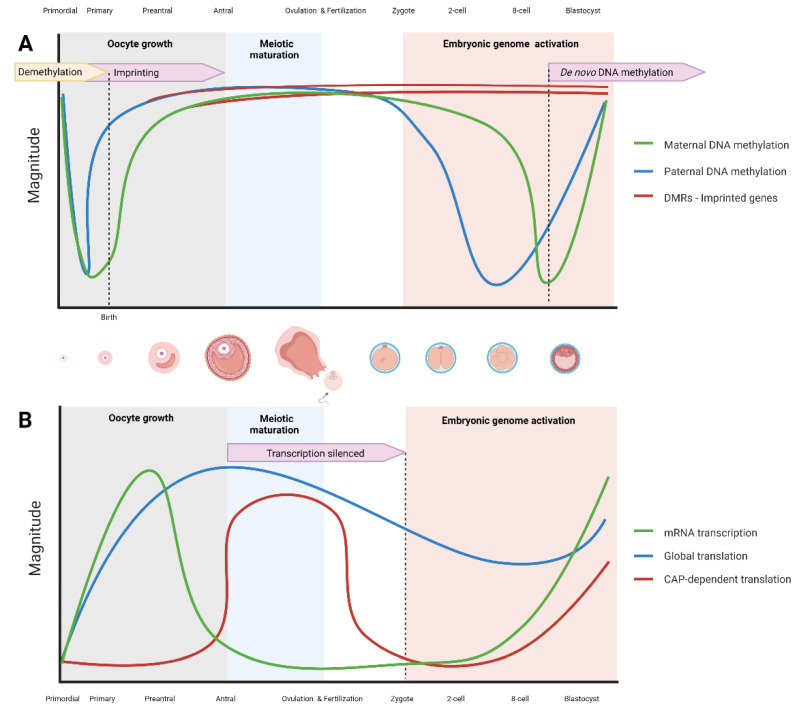
Dynamic DNA methylation and protein translation changes in human oogenesis and early embryogenesis: (**A**) Prenatal DNA demethylation in primordial germ cells (PGC) is followed by de novo DNA methylation, which occurs earlier in males than females. Genomic imprinting loci consisting of DMR (differentially methylated regions) maintain their methylation status despite the important genome-wide DNA demethylation in pre-implantation embryos [1]. (**B**) Oocytes are transcriptionally active during oocyte growth with rapid decline and silencing throughout meiotic maturation [2]. According to the new evidence human embryonic genome activation is initiated at the zygotic stage, but the transcriptional activity remains low until the 8-cell stage [3]. Correct regulation of CAP-dependent translation is a key process in meiotic maturation despite continuous decrease in global translational activity from oocytes to zygotes. The image was created with BioRender.com.

DNA methylation is the most studied epigenetic mechanism and plays a key role in transcriptional repression/activation (Figure 1 and Figure 2).

In vertebrates, DNA cytosine methylation is realized by the addition of a methyl group onto the fifth carbon of cytosine residue within cytosine-phosphate-guanin (CpG) sites, where a cytosine is followed by a guanine in the 5′ → 3′ direction of the DNA linear sequence. DNA methylation is functionally associated with gene silencing and is for the most part limited to CpG islands, i.e., areas rich in CpG dinucleotides that are typically located within and nearby sites of about 40% of mammalian gene promoters [4,5]. As the CpG dinucleotides are methylated symmetrically on both DNA strands, their methylation can be heritable during cell division [4].

DNA is methylated by methyltransferases (DNMTs), and the methyl group for methylation is provided by S-adenosylmethionine [6]. DNA methylation relies on the folate-methyl metabolic pathway that supplies the essential methyl groups [7]. There are two distinct types of DNA methylation. During cell division, DNA replication creates hemi-methylated DNA where CpG dinucleotides are methylated only on the original parent strand and methylation is absent from the newly synthesized daughter strand. Subsequently, the methylation maintenance methyltransferase DNMT1 methylates the unmethylated DNA strand [8]. In contrast, de novo methylation of un-methylated double stranded DNA is ensured by DNMT3A and DNMT3B along with their coactivator DNMT3L [9,10].

DNA cytosine methylation can affect DNA activity and when it occurs in a gene promoter, increased DNA methylation leads to a decrease in chromatin accessibility. Subsequently, gene transcription is repressed by recruiting gene-silencing repressive proteins to the methylated region, such as methyl-CpG-binding protein 2 (MeCP2) and methyl-binding-domain (MBD) proteins [11,12]. Methylated DNA also presents a spatial obstacle preventing binding of transcription factors and proteins to gene promoters [13,14]. However, methylation of CpGs over gene bodies is related to both active and repressed transcription according to the tissue in which it occurs [15,16]. Evidently, DNA methylation has dual roles, both inhibitory and permissive, depending on the genomic region.

Epigenetic modifications to RNA, the field of epitranscriptomics, have changed our perception on how epigenetics can directly modulate the translation and stability of mRNAs within oocytes and embryos. There are over 150 post-transcriptional mRNA transcript modifications in eukaryotes, but the most prevalent is the N^6^-methyladenosine (m^6^A) methylation (Figure 1) [17]. Generally, m^6^A transcript methylation is present ubiquitously in every cell and across multiple species. Translation-related m^6^A modification occurs within the 3′ UTR of mRNA. The interaction between the m^6^A reader and translation initiation factors is needed for cap-dependent translation [18]. m^6^A were also found to be capable of governing translation in a cap-independent manner by modifications within internal ribosome entry sites (IRES) [19] or 5′ UTR sequences [20]. A direct link between greater polysome occupancy and the presence of m^6^A methylation on mRNAs was demonstrated in yeast [21] and HeLa cells [18].

Epigenetic regulation by non-coding RNAs (ncRNAs) recently gained more attention in developmental biology literature. ncRNAs represent RNA molecules that do not code for proteins but have a regulatory role at pre- and post-transcriptional levels [22]. Generally, both short and long ncRNAs do exist. Among the smaller ones are small-interfering RNA (siRNA), microRNA (miRNA), piwi-interacting RNA (piRNA), followed by long ncRNA (lncRNA) (Figure 1). As ncRNAs are also implicated in histone modification and DNA methylation, it has been suggested that gene silencing and upregulation by ncRNAs is a part of the epigenetic mechanism [23,24]. The importance of ncRNAs in regulating primordial germ cell specification, spermatogenesis, and oogenesis has been highlighted (reviewed in [25,26]). ncRNAs are intensely expressed in the early human embryo and their role in early human development remains to be fully investigated. It has been suggested that future studies of the ncRNAs role could expand to the field of ART optimization [27,28].

## 2. Epigenetics of Germ Cells Development

### 2.1. Epigenetic Reprogramming

Two different waves of epigenetic reprogramming occur in gametes and the early embryo, the first one during gametogenesis and the second in the preimplantation embryos (Figure 2) [29,30]. During mammalian gametogenesis, pre-existing epigenetic marks are globally deleted in the primordial germ cells. During sex determination in the developing fetus, the global DNA methylation remains at very low levels in both male and female primordial germ cells [31]. However, de novo methylation establishment proceeds in different manners in male and female germlines. In the male gonocytes, a rapid increase of methylation is initiated during embryo development, and the methylome of male germ cells is fully established before the birth [32,33]. In female germ cells, DNA methylation is re-established during the postnatal oocyte growth phase and is dependent on the functional interaction of the DNA methyltransferase Dnmt3a and the Dnmt3-like protein (Dnmt3L) [34,35]. DNA de novo methylation is completed when oocytes reach the germinal vesicle stage [1,36]. After a period of intense activity during oocyte growth, transcription is ceased when the oocyte becomes arrested at the prophase of meiosis I, and this arrest is released after a preovulatory hormonal surge of gonadotropins [37]. It has been proposed that the phenomenon of meiotic arrest possibly protects oocytes from oxidative stress and DNA damage [38]. During oogenesis, the oocyte development and competence is not dependent on DNA methylation as oocytes with genetically ablated DNA methylation were successfully fertilized, and subsequent embryonic development progressed until the mid-gestation stage [39,40]. It has been suggested that methylation of CpG islands in gametes is not fully related to genomic imprinting but is an important factor in the regulation of gene expression in preimplantation embryos [34].

### 2.2. Histone Deacetylation in Oocyte Maturation and Energy Metabolism

Histones are positively charged proteins, which form the integral part of the chromatin core. The octamer core is composed of four histone variants—H2A, H2B, H3, and H4—with connecting histone H1 [41]. They are essential for packaging the DNA into superstructures, making it inaccessible for DNA-binding proteins to bind it and recruit further transcription or regulation machinery. Important modification regulating histone interactions are classified into methylations, acetylations, phosphorylations, ubiquitinations, and PolyADP ribosylations (Figure 1) [41]. Particularly, the regulation of histone acetylations is relevant for the final moments of oocyte maturation.

Histone deacetylases (HDACs) are enzymes that regulate a wide range of biological processes by removing acetyl groups from lysine ϵ-amino groups, not only on histones but also on many other proteins [42]. In particular, meiotic progression was discovered to be dependent on HDACs activity progression in mice. Histone acetylation of lysine residues was substantially reduced upon meiotic resumption in mouse oocytes [43]. Maintenance of genome integrity and chromatin structure is controlled by HDAC3. In meiosis, HDAC3 is located on the spindle. HDAC3 knockdown experiments on mice revealed defects in chromosome alignment, spindle structure, and microtubule-kinetochore attachment (MT-K) [44]. Suppression of HDAC3 activity in porcine oocytes led to similar phenotypes as in mice, spindle defects, chromosomal congression failure, and meiosis inhibition [45]. Disruption of oocyte maturation by the selective HDAC6 inhibitor tubastatin-A induced asymmetric division in maturing oocytes, failure to extrude the first polar body [46], increased α-tubulin acetylation, and incorrect MT-K attachment as seen in HDAC3 [47]. Recently, primordial follicle activation by mTOR signalling was associated with decreased HDAC6 activity. This finding may be of importance for the management of premature ovarian failure (POF) as an alternative approach to primordial follicle in vitro activation (IVA) [48]. HDAC8 has a similarly indispensable role as HDAC6 and is located on spindle poles. Its absence led to the defective recruitment of γ-tubulin and consequently caused aberrant spindle morphology and chromosome misalignment in mice [49] and pigs [50]. The activity of HDAC1/2 in embryos is required for proper DNA methylation, cell lineage development, and transformation from morula to blastocyst [51]. HDAC3 is closely connected with HDAC 11. Its inhibition increased the acetylation level of α-tubulin [45,52], significantly impaired the course of meiosis in mouse oocytes, and disrupted kinetochore–microtubule attachment and spindle assembly checkpoint [52], which resulted in abnormal spindle organization and chromosome misalignment.

The regulation of histone acetylation in oocyte meiosis is also reliant upon specific NAD^+^-dependent HDACs, silent information regulator 2 (Sir2) proteins (“sirtuins”) that belong to a seven-member family of deacetylases involved in the deacetylation of histones as well as nonhistone proteins. Moreover, sirtuins are employed in the regulation of metabolism, inflammation, and oxidative stress. [53]. SIRT1 plays a role in the activation of primordial follicles in a deacetylase-independent manner [54] and was reported to slow down the aging-related decrease of oocyte quality, which may under in vitro laboratory conditions impact oocyte maturation [55]. SIRT1 overexpression decreased H3 histone methylation and acetylation in post-ovulatory aged mouse oocytes as well as decreased aging related reactive oxygen species (ROS), spindle abnormalities, and mitochondrial dysfunction [56].

The SIRT2 impaired gap junctional communication during in vitro maturation of bovine oocytes by phosphorylation of connexin-43 [57] is another sirtuin activity. In human serum samples from IVF patients, a basal SIRT2 level was proposed as a pregnancy outcome predictor in combination with age, anti-Mullerian hormone (AMH), and antral follicle count (AFC) [58].

At the GV stage of mouse oocytes, SIRT7 is located within the nucleus, then upon meiotic resumption it is dispersed in the cytoplasm, with the highest SIRT7 concentration occurring around the chromosomes. SIRT7 knockdown compromised mitochondria function, significantly decreased ATP levels, and increased ROS [59]. SIRT4 exhibited similar phenotype to SIRT7 [60]. On the other hand, SIRT6 interacts with chromatin proteins and is employed in DNA double-strand break (DSB) repair mechanisms. Its knockdown in early mouse embryos shortened telomeres and caused an increase in DNA damage [61].

Sirtuins are also implicated in the regulation of energy metabolism and stress resistance; particularly, SIRT3, SIRT4, and SIRT5 mainly localize in mitochondria [62]. SIRT1 and SIRT3 have been revealed to play a crucial role in ensuring protection against oxidative stress in oocytes, granulosa cells, and early embryos [63]. It has been reported that the SIRT1 anti-oxidative stress effect in mouse oocytes is attenuated during aging [64]. Recently, a decrease of ovarian reserve in mice was linked to SIRT1-related changes in mitochondrial oxidative phosphorylation [65]. Additionally, a protective role of SIRT3 against oxidative stress was revealed in preimplantation mouse embryos [66]. A correlation between the decreased expression of SIRT3 and lower embryonic developmental competence was found in human in vitro cultured embryos [67].

### 2.3. Fertilization & Mitochondria

Mitochondria play a major role in providing each cell with energy by generating adenosine triphosphate (ATP) through electron transport-linked oxidative phosphorylation (OXPHOS) [68]. Since the aerobic respiratory pathway in eukaryotic cells is the only system that fully relies on mitochondria function [69], any mutations in mtDNA or nuclear-encoded mitochondrial genes can result in mitochondrial dysfunction that induces a variety of pathologies and contributes to an abnormal aging process [70,71]. Mitochondria are the prominent source energy for successful oocyte and sperm biogenesis and function. This dependence is due to the high energy demand for the support of proper chromosome segregation and the fertilization process [72,73]. In mammalian oocytes, a sufficient mtDNA copy number is essential to promote fertilization and early embryo development. Human oocytes with fewer than 100,000 copies of mtDNA evince a significantly lower fertilization rate than oocytes with more than 150,000 copies [74,75]. In vertebrates, inheritance of mitochondria is maternal as the paternal mitochondria of sperm origin are eliminated during early embryo development [76,77].

Mitochondrial activity is an indicator of oocyte developmental competence [78]. Oocyte maturation and early embryo development depend on ATP derived mainly from mitochondrial oxidative phosphorylation [79,80]. Events such as the formation and maintenance of the meiotic spindle are also dependent on mitochondrial function [81]. Deficiency of ATP and low mtDNA copy number are associated with poor oocyte quality, retarded embryo development, aneuploidy, and decreased implantation and placentation rates [82,83]. mtDNA has a considerably higher mutation rate than the nuclear genome, and it is assumed that mtDNA is prone to oxidative damage induced by reactive oxygen species [84]. Mitochondrial dysfunction and deficiency of mitochondria-derived ATP provoked by oxidative stress induces spindle disruption in MII mouse oocytes [85]. Mutations of mtDNA can cause a set of physical and cognitive disabilities, including pathologies of the nervous and muscular systems. However, progress has been made recently in the field of inherited mitochondrial disease and therapeutic approaches, such as the development of pre-implantation genetic screening and mitochondrial replacement therapy [86,87].

A decrease of mitochondrial number, function, and mtDNA quantity affect the viability of oocytes and female fertility [88,89]. Advanced maternal age is associated with a reduction of ATP production that leads to decreased metabolic activity and can negatively affect cell cycle regulation, meiotic spindle formation, chromosome segregation, fertilization, embryo development, and implantation [72,90,91]. An increased expression of the mitochondrial unfolded protein response gene *Hspd1* in GV oocytes of PMSG-treated aged mice reflects the mitochondrial stress caused by advanced age [88]. In ovaries of aged mice, a decreased mRNA expression of mitochondrial antioxidants *Prdx3* and *Txn2* was reported [92]. An increased expression of the mitochondrial antioxidant *TXN2* gene and mitochondrial transcription factor *TFAM* gene in cumulus cells of unstimulated aged cattle was detected [93]. To enhance the fertilization rate of aged oocytes, the technique of mitochondrial supplementation can be applied. For this purpose, it is possible to use the method of either partial or total cytoplasm transfer from donor to recipient oocyte [94,95].

### 2.4. Genomic Imprinting in Early Embryo

Genomic imprinting is defined as a monoallelic parent-of-origin-dependent gene expression in offspring and consists in differential methylation inherited from the gametes when one parental copy of the gene is silenced while the other parental allele is expressed [96]. DNA methylation is one of the epigenetic changes regulating the expression of imprinted genes during early development. It is an epigenetic process that involves DNA methylation and histone methylation without altering the genetic sequence. These epigenetic marks are established (“imprinted”) in the germline (sperm or egg cells) of the parents and are maintained through mitotic cell divisions in the somatic cells of an organism [14]. Although extensive nuclear reprogramming occurs in preimplantation embryos, the methylation of imprints acquired through gametogenesis escapes from this global epigenetic reprogramming and persists in preimplantation embryos (Figure 2) [9,97]. The methylation of the imprinted genes is thus preserved and then transmitted to the offspring. As of 2019, around 260 imprinted genes have been identified in mice and 230 in humans [98]. Imprinted genes are involved in the regulation of embryonic growth, placental function, postnatal growth, and neurobehavioral processes [99,100]. In humans, abnormal expression of some imprinted genes has been related to numerous diseases, developmental abnormalities, and malignant tumours [101,102,103]. Although DNA methylation is a key player in genomic imprinting through the establishment of imprinted marks on either paternal or maternal alleles (Figure 2), the genomic imprinting process is significantly influenced also by histone modifications and non-coding RNA [104,105,106].

### 2.5. Embryonic Genome Activation

During early embryogenesis between fertilization and implantation, parental DNA is subjected to rapid and extensive demethylation, and consequently epigenetic information inherited from the gametes is vastly reset in the preimplantation embryos [9,107]. In human embryos, a sharp decrease of paternal DNA methylation occurs between fertilization and the two-cell stage; however, the decrease of maternal DNA methylation is milder (Figure 2) [108]. The DNA methylation level is also decreased during zygotic activation in mice, bovine, and goat preimplantation embryos [109,110,111]. In mice, the most intense demethylation occurs in the zygotes, and gradual demethylation continues until the blastocyst stage [112]. The newly activated DNA demethylation that occurs during the early pronuclear stage precedes the increase of DNA replication indicating that DNA demethylation in the early zygote is independent of DNA replication [113].

The early human embryo consists of a large number of transposable elements (TE) that could be a potential cause of gene rearrangements, mutations, deletions, or duplications. Therefore, as a precaution, silencing of these evolutionary younger TE is ensured by DNA methylation or histone modifications [108].

Nearly all methyl groups are removed from the paternal-origin DNA immediately after fertilization [97]. Methylation of maternal-origin DNA is diluted with each round of replication and results in a substantial decrease of DNA methylation during the morula stage [114]. Global epigenetic reprogramming occurs in the early embryo when DNA demethylation is at the highest levels in the early blastocyst stage [115]. The genome-wide erasure of CpG methylation is more profound in early embryos from superovulated mice when compared to embryos from naturally mating control mice [116].

### 2.6. Intrauterine Epigenetic Inputs

Subsequent reinitiation of DNA methylation occurs in the blastocyst stage in the cells of the inner cell mass and establishment of new methylation marks continues during fetal development [97]. During post-implantation development the activity of DNMT3A and DNMT3B together with their coactivator DNMT3L are essential to establish the characteristic methylation profile in the developing embryo [117]. DNA methylation provides an epigenetic regulatory mechanism protecting the differentiating cells from regression to the undifferentiated state [115]. In post-implantation embryos, DNA methylation is an integral of epigenetic marks in the majority of embryonic tissues and persists in somatic tissues during the lifespan of adults [118]. Mice embryos with insufficient DNA methylation activity die at mid-gestation as a consequence of genome-wide demethylation [119].

## 3. Epigenetics within Translational Regulation: The Oocyte to Embryo Story

### 3.1. Active Transcription Fuels Maturing Oocyte

Primordial germ cells (PGCs) that are produced from female germ cells undergo mitosis, forming oogonia and during subsequent oogenesis, the oogonia become primary oocytes. Every oocyte originates from primordial germ cells (PGCs). PGCs migrate in utero into a future ovary and go through repeated mitotic cycles to form nests of germ cell syncytia [120]. All female mouse primordial germ cells are connected by intercellular bridges in the ovaries at embryonic day 11.5 to 17.5 and form synchronously dividing interconnected cysts or syncytia of up to 30 germ cells [121,122]. Following the homologous recombination and formation of cytoplasmic bridges, oocyte nuclei are arrested at the diplotene stage of meiotic prophase I. Following birth, germ cell nests are dispersed along with the invasion of pre-granulosa cells [121]. Individual primordial follicles made of primary oocytes enclosed by a basal layer of flattened granulosa cells are formed. However, a substantial amount of primordial follicles undergo atresia, and one of the proposed functions for follicular atresia is the selection of follicles containing oocytes of the highest developmental potential [123,124]. Activation of the primordial follicle that starts at prepubertal stage and extends throughout the reproductive life is cross-regulated by key transcriptional factors (FIGLA, LHX8, and SOHLH1) that cooperate on common downstream pathways in folliculogenesis [125]. If a primordial follicle is activated, it does so via the binding of Kit Ligand from granulosa cells (KL) onto a Kit receptor present on the oocyte and theca cells [126]. This Kit-KL system is connected downstream by a PI3K/Akt pathway [127]. A feedback loop is further secured by oocyte-secreted factors (OSFs) such as BMP-15 or GDF-9 [128,129]. As the oocyte grows further, macromolecules, proteins, and transcripts are rapidly accumulating within. Experiments on bovine oocytes have shown that a substantial amount of mRNA transcripts are capable of transport from granulosa cells into the oocyte via gap-junctions. These connections between the oocyte and cumulus cells were subsequently named as transzonal projections (TZPs) [130,131]. Once the oocyte reaches the fully grown germinal vesicle stage (GV), the transcription in the GV nucleus is ceased. Accumulated mRNAs are more stable than those present in somatic cells [132]. Further, during the MI/MII transition, the oocyte is dependent on the effective utilization of stored transcripts and proteins [133,134], which needs to be tightly regulated based on the metabolic requirement, nutrient availability, and presence of environmental stress. Fine tuning of oocyte meiosis and early embryo development is ensured by translational regulation.

### 3.2. Translational Regulation, the Key for Oocyte Success

The regulation of translation is orchestrated by many mechanisms, ranging from modulating polyA tail length, modifying mRNA post-transcriptionally, regulating interactions between proteins, degrading stored RNAs, clustering RNAs into ribonucleoproteins (RNPs), up to the 5′-terminal oligopyrimidine (TOP) motif mRNA regulation under stress from nutrient or oxygen deprivation [135]. The best known regulation mechanism of selective mRNA translation is the 3′ UTR polyadenylation, which works for about 70% of the oocyte’s mRNAs [136]. PolyA tail length positively correlates with ribosome occupancy. The ribosome loading itself is regulated by the CPE binding protein 1 (CPEB1) and deleted-in azoospermia such as (DAZL) binding to the cytoplasmic polyadenylation element (CPE) on the 3′ UTR of mRNA. Their absence compromises mouse oocyte meiotic maturation and MII oocyte development by dysregulating effector proteins, polyA binding protein 1 (PABP1), and polyA specific ribonuclease (PARN) responsible for mRNA polyadenylation and deadenylation respectively [137].

As oocyte meiosis is progressing, global translation decreases (Figure 2). However, translation of m7G capped mRNAs is mainly regulated by the mTOR/S6K1/4E-BP1 signalling (mTORC1 pathway) [2,138]. The main player here, the mammalian target of rapamycin (mTOR), a serine/threonine protein kinase, regulates diverse cellular functions (reviewed in [139]). It has been documented that mTOR activation and the protein synthesis initiation is influenced by the activity of cyclin-dependent kinase 1 [140]. mTOR is responsible for translational regulation of capped mRNAs containing a TOP motif by recruiting an eukaryotic initiation factor 4F (eIF4F) [2] and RNA-binding protein LARP1 [135]. Oocyte-specific conditional knockout of mTOR severely affected folliculogenesis. Deleting mTOR in meiotic maturation changed the oocyte’s proteome composition, caused spindle instability, aneuploidy, and failure to form a methaphase II equatorial plane [141].

Translatability of mRNAs is also indirectly regulated by the formation of superstructures with proteins into RNP cytosolic granules. In such a way, translation, degradation, and storage of transcripts can be controlled simply by regulating their physical availability to the translational or degradational machinery. RNP granules commonly found in oocytes and embryos are stress granules or p-bodies. Stress granules are formed upon nutritional deprivation, heat, or oxidative stress by liquid-liquid phase separation [142]. P- bodies, on the other hand, are engaged in the storage of mRNAs with regulatory functions, previously thought to play a role in RNA decay [143]. Continuous degradation of stored RNA expressed during early stages of oogenesis occurring in meiosis and during early embryo development is preventing them from being inherited by simple eradication of redundant RNAs [144].

### 3.3. Epitranscriptomics—Translational Regulation by mRNA Methylation

Not only oocyte translational regulation is key for meiotic maturation, but also the recently discovered regulation by the epitranscriptomic m^6^A mRNA methylation [145,146]. Earlier studies in Xenopus oocytes found m^6^A methylation to inhibit mRNA recruitment for translation. Key cell cycle and translation-related transcripts in Xenopus were demethylated in order to become translated [145]. However, a study on porcine oocytes showed rapid accumulation of m^6^A methylated transcripts inside the ooplasm as meiotic maturation progressed. Inhibition of m^6^A methylation by cycloleucine, a specific inhibitor of adenosyl-transferase, impaired oocyte maturation and further development [147]. A role of m^6^A mRNA methylation during meiotic maturation and maternal to zygotic transition has been confirmed in the mouse model [146], and proper regulation of m^6^A mRNA methylation was shown to be crucial for both preimplantation [148] as well as in utero [149] embryo development.

Recent evidence stressed the importance of m^6^A mRNA methylation in the development of fully matured and developmentally competent oocytes and early embryos. Epitranscriptomic m^6^A RNA methylation is an ubiquitous and reversible process orchestrated by methyltransferases (“writers”), binding proteins (“readers”) and demethylases (“erasers”) [150,151].

Three major epitranscriptomic writers exist: METTL3, METTL14, and METTL3 adapter—WTAP [51,152]. The methyltransferase complex METTL3 was studied in detail on mouse oocytes. Transient knockdown of METTL3 by RNAi led to a substantial decrease of mRNA translation efficiency, low oocyte maturation rate, problems with maternal to zygotic transition [146], and inability to form blastocysts [148]. METTL3 was found to play a role in folliculogenesis, ovulation, maintenance of DNA integrity, and preimplantation development [153]. The same enzyme is engaged in the angiogenesis of atherosclerotic mouse model embryos by upregulating vascular-endothelial growth factor (VEGF) [154] through m^6^A [155]. In zebrafish embryos, METTL3 regulated PHLPP2/mTOR-AKT signalling [156]. The recently published data have shown METTL3 direct interaction with the known p53 transcription factor that enhanced p53 stability and together cooperatively modified p53 targeted RNAs by m^6^A upon DNA damage [154]; METTL14 was found to be needed for embryonic post-implantation epiblast formation [149]. Further, data have shown the importance of methyltransferase KIAA1429 for folliculogenesis and oocyte development, as the KIAA1429 conditional knockout mice produced severe defects in oocyte growth, alterations in OSFs expression, and an inability to undergo nuclear envelope breakdown (NEBD) [157].

Many proteins can act as m^6^A readers. The most known are YTH domain containing family proteins (YTHDF1,2,3) [158] and IGF2BP1 protein regulating JAK2/STAT3 signalling [159], as well as many others. YTHDF1 promotes active translation in HeLa cells by interconnecting m^6^A mRNA transcripts with translation initiation factors, ribosomes, or stress granules [18]. YTHDF3 was shown in HeLa cells to enhance YTHDF1 upregulation of translation as well as the promotion of RNA decay via YTHDF2 [160]. The YTHDF2 reader in mice is responsible for the maintenance of correct gene dosage by utilizing RNA degradation machinery. This is coupled with the activation of CNOT7 deadenylase and DCP1A, DCP2 decapping enzymes. However, the most recent study on HeLa concluded that all YTHDF1,2,3 readers have common core sites and act together on selective m^6^A mRNA degradation via CCR4-NOT deadenylation complex [158]. Female YTHDF2 double knockout mice are infertile and show cytokinesis defects in early zygotic development. Nevertheless, YTHDF2 double knockout oocytes are capable of ovulation and fertilization [161]. Additional enzymes do exist that relay m^6^A regulation onto common physiologically important pathways. For example, recently discovered m^6^A reader activity in Fragile-X mental retardation protein (FMRP) is important for the maternal RNA decay in Drosophilla embryos. FMRP used m^6^A tagged mRNA transcripts for their sequestering into FMRP granules. [162]. FMRP was also shown to create granules at the onset of meiosis in human fetal ovaries, suggesting its importance in the translational regulation of oocyte maturation [163]. This was supported by the detection of FMRP in all stages of mouse oocyte meiotic maturation and its rapid decline in two cell embryos [26]. Therefore, m^6^A methylation could both directly and indirectly regulate the translation of certain mRNAs.

The removal of m^6^A methylation marks from transcripts is done by two main demethylases (“erasers”), fat mass- and obesity-associated (FTO), and α-ketoglutarate-dependent dioxygenase alkB homolog 5 (ALKBH5) [164]. A decrease of FTO expression with age was shown to increase m^6^A methylation in aged mouse ovaries and human granulosa cells of elderly patients [162]. The same decrease of FTO expression followed by an increase in m^6^A methylation was observed in ovarian tissues from premature ovarian failure (POF) patients and POF model mice [165]. Therefore, proper regulation of m^6^A methylation is one of the factors to ensure follicular developmental competence. Decreased m^6^A methylation in placental tissues of patients suffering from recurrent miscarriage caused by the upregulation of the second eraser, ALKB5H demethylase, revealed that in such endometrium the trophoblast is unable to nidate [166].

## 4. Translation of Epigenetics into ART

**Figure 3:**
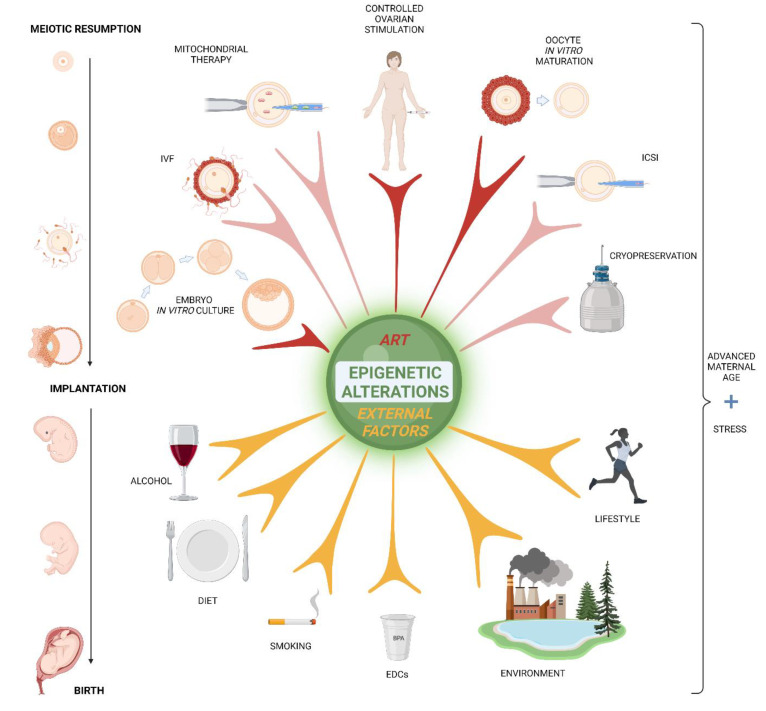
Assisted reproduction technology (ART) and external factors involved in epigenetic alterations: visualization of ART procedures employed in the process of oocyte meiotic maturation and early embryo development with proven (red arrows) or insignificant (pale red arrows) impact on the epigenome. External factors influencing epigenetics of post-implantation in utero embryo and fetal development (yellow arrows) are divided among nutritional (alcohol, diet) and lifestyle factors (advanced age, smoking, living environment, endocrine-disrupting chemicals (EDCs)). The image was created with BioRender.com.

Assisted reproduction techniques (ARTs) are widely applied in the field of human reproduction (Figure 3) and animal breeding. Exposure to ART results in a decreased developmental competence of fertilized mouse oocytes, partially due to the induction of epigenetic changes [167] (Table 1).

In humans, no significant epigenetic changes were found between regular pregnancy and ART pregnancy in newborns, however only a few key imprinted genes were analyzed in a small cohort of patients [190]. Recently, epigenetic imprinting-related disorders were demonstrated on mouse models and also observed in ART newborns like Prader–Willi syndrome (PWS), Silver–Russell syndrome (SRS), Beckwith–Wiedemann syndrome (BWS), and Angelman syndrome (AS) [191]. The long-term epigenetic effects of ART still await evaluation. Needless to say, more extensive follow-up of children born from ART embryos should be carried out. Moreover, further investigations into the epigenetic impact of ART methodology on cultured oocytes and embryos should be done.

### 4.1. Hormonal Stimulation

Superovulation or the clinical term controlled ovarian stimulation (COS) is applied to statistically increase the chances of acquiring blastocyst stage embryos compared to normal ovulation and increase the likelihood of a successful pregnancy. The basis of COS is the stimulation by a recombinant follicle stimulating hormone (rFSH) and the ovulation trigger, human chorionic gonadotropin (hCG). A co-administration with gonadotropin-releasing hormone (GnRH) agonist or antagonist is needed to avoid premature ovulation.

Increased incidence of chromosomal aneuploidy in human COS oocytes was associated with changes in DNA methylation [168]. COS was also linked to embryo development retardation [192] and negative effects on child health [193,194,195] A loss of genomic imprinting, particularly associated with the overgrowth Beckwith–Wiedemann (BWS) syndrome was reported in bovine fetal tissues. Demethylation of imprinted genes was observed in other bovine genome loci [196].

Additionally, protein translation was affected using the mechanisms triggered by superovulation. The mRNA expression of two critical players in translational regulation of stored maternal mRNAs, the embryonic poly(A)-binding protein (ePAB), and the poly(A)-binding protein cytoplasmic 1 (PABPC1), was modified in oocytes and two-cell embryos [170]. After IVM oocyte culture in the presence of rFSH, a decreased global translation was observed in mouse, bovine, and porcine models including humans [197]. rFSH may compromise regulation of specific translatome essential for oocyte maturation and early embryo development. Therefore, the use and dosage of recombinant hormones in the conventional IVM should be thoroughly evaluated.

Superovulation in mouse oocytes and early embryos alters DNA methylation [198] and expression of methyltransferases [169] and impairs methylation of genes involved in glucose metabolism, nervous system development, cell cycle, cell proliferation, and mRNA processing [172]. Disrupted DNA methylation of imprinted loci in mouse blastocysts affected, for example, a body-weight-limiting H19 gene [173], and was more frequent at higher hormonal superovulation dosages [171]. Superovulation caused imprinting defects leading to embryonic abnormalities and higher mortality [174,199]. Detected epigenetic differences in DNA methylation between superovulated and naturally ovulated oocytes suggested that superovulation also recruits growing oocytes with incomplete epigenetic maturation [172]

### 4.2. Oocyte In Vitro Maturation

When hormonal stimulation does not produce a satisfactory number of matured MII oocytes, in vitro maturation (IVM) is considered. IVM is based on the retrieval of fully grown cumulus enclosed GV oocyte complexes (COC) from ovarian follicles. COCs are meiotically matured by the IVM in the presence of rFSH and LH (rLH) to produce MII oocytes. The conventional IVM technology is understandably inferior to standard COS with maturation in vivo.

As just mentioned, rFSH promoted a decrease of global proteosynthesis marker, the radioactively labelled 35S methionine, in mouse, bovine, porcine, and human IVM oocytes [197]. The use of recombinant hormones in the IVM media is one of the factors affecting proper translational regulation and proteosynthesis during maturation.

A hypothesis was proposed that the resumption of meiosis upon COC ovarian puncture (OPU) is premature and could also have a negative impact on IVM quality. In response to that, an alternative approach called capacitation IVM (CAPA-IVM) was devised [200]. This experimental ART maintains high cAMP concentration within the in vitro cultured human COC by stimulating cumulus cells with rFSH, insulin, estradiol, and C-natriuretic peptide, thus indirectly inhibiting the NEBD in oocytes for about 24 h. The recent clinical study resulted in a live birth rate after the first embryo transfer of 35.2% for CAPA-IVM compared to 43.2% for standard IVF control [200]. It can be hypothesized that CAPA-IVM has a direct downstream impact on translational regulation as it gives the oocytes more time to equilibrate before meiotic maturation. These promising results from CAPA-IVM deserve further investigation, particularly the clarification of underlying molecular mechanisms and epigenetic regulation.

IVM quality is indeed determined by epigenetic m^6^A transcript methylation or histone deacetylations. Numerous histone modifications are employed in oocyte meiotic maturation, ranging from most profound deacetylation to methylation, phosphorylation, ubiquitination, and SUMOylation as reviewed by He et al., 2021 [41]. General histone deacetylation was compromised throughout meiosis in aged human oocytes. The residual acetylation correlated with chromosome misalignment linked to aneuploidy [181]. Moreover, human IVM oocytes with reduced HDAC1 expression also exhibited MII spindle abnormalities. Comparatively, more HDAC1 transcripts were present in in vivo (IVO) matured oocytes than IVM [201].

Improved IVM technology could also utilize the discussed sirtuin deacetylase family as recently reviewed [53]. Authors of the review detected sirtuins application for the management of aging and stress related syndromes, PCOS, diabetes, or endometriosis, and concluded the beneficial improvement of the energy balance and ROS protection. Decreased SIRT3 expression for example, correlated with lower developmental competence in human in vitro cultured embryos, which was attributed to defective mitochondrial biogenesis [67]. Recently, basal human serum SIRT2 level was suggested as a novel biomarker of ART outcome [202]. This kind of IVM therapy is promising, however further investigation is needed to determine their best delivery, dosage, combination, and mode of action.

The IVM approach is most applied for the management of polycystic ovarian syndrome (PCOS) which is the most common endocrine disorder in 6–20% of women of reproductive age accompanied by oligoovulation and/or anovulation. PCOS is a multifactorial syndrome with strong epigenetic inheritance, where external environmental, lifestyle, and dietary factors play a significant role [203].

**Figure 4:**
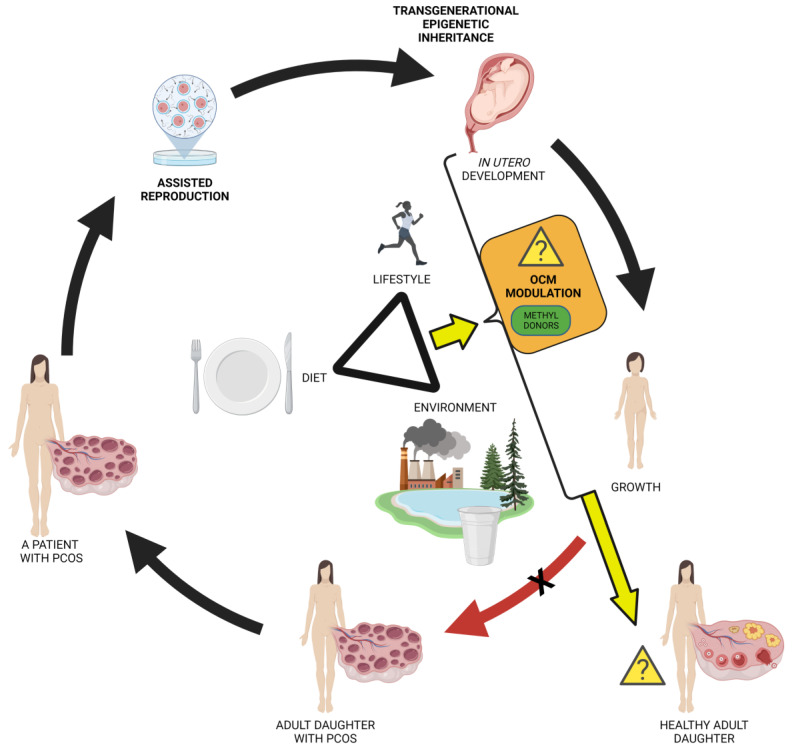
**Transgenerational epigenetic inheritance and the therapy (the polycystic ovary syndrome (PCOS) case:** Recent evidence showed that acquired epigenetic changes can be inherited between generations. One such example is the PCOS, which presents the main cause of infertility in humans. PCOS patients are usually managed by assisted reproduction techniques (ART), but there is high probability of transmission from mother to daughter. DNA hypomethylation in PCOS is also influenced by external factors such as nutrition, living environment, or lifestyle [205]. The nutritional supplementation with one carbon metabolism (OCM) compound, the methyl donor S-adenosylmethionine (SAM) could possibly have a therapeutic potential to mitigate or alleviate PCOS in humans [206]. The image was created with BioRender.com.

PCOS infertility is managed through IVF centres. Recent evidence suggests intergenerational epigenetic inheritance of this syndrome [204]. The puzzling question arises whether PCOS is trans-generationally inherited through offspring, who would again become clients of IVF clinics (Figure 4). Instead, could PCOS be cured by managing mentioned external epigenetic factors rather than just relayed onto the next generation by IVF?

### 4.3. Mitochondrial Therapy

Oocyte developmental competence is tightly connected with energy availability. Meiotic maturation, fertilization, and embryonic genome activation are all energetically demanding processes depending on essential mitochondrial ATP production through OXPHOS. Metabolic disorders and aging are coupled with oocytes of inferior quality, which is largely attributable to energy production in the form of ATP by mitochondria as reviewed by [207]. mtDNA copy number in human oocytes range from 20,000–600,000 [79,208] and was found to decline with increasing maternal age [209]. The ART solution to this trend in our population is to apply cutting-edge mitochondrial therapies and take advantage of enucleated donor oocytes in techniques such as Cytoplasmic Transfer (CT), Maternal Spindle Transfer (MST), first Polar Body Transfer (PB1T), or in case of zygotes, Pronuclear Transfer (PNT). Nuclear transfer in GVs has been described, but due to complete zygote arrest of such maturated and fertilized mouse model oocytes, is not further considered for clinical applications (reviewed by [210]).

CT of about 10–15% of donor oocyte cytoplasm into one of the two sibling oocytes of an aged patient increased their developmental competence. Newborns from such treated oocytes were considered being healthy [95].

The MST method is based on the removal of the MII spindle from a healthy donor oocyte. In the same way, MII spindle is transferred from the patient’s affected oocyte into a healthy enucleated donor oocyte [211,212]. The first MST in human oocytes had low zygote survival ability, but with comparable blastocyst rates [211]. In 2017, MST was used for the first time therapeutically in the management of Leigh syndrome, which affects mtDNA and mitochondria [213]. Following a live birth, the load of mutated mtDNA was estimated to be below 10%. Concerns about MST introducing new epigenetic changes in donor oocytes were negligible as revealed by analysis of gene expression, which found no significant differences [214].

Whole genome RNA-sequencing of PNT derived blastocysts also found no major epigenetic differences [215]. An inferior method to MST, the PB1T method successfully reconstituted spindle in about 67% of enucleated donor oocytes but following fertilization only about half of the produced viable zygotes were capable of blastocyst development [216,217]. First polar bodies (PB1) were shown to mirror the methylomes of the oocytes they originated from, and therefore PB1s could have more of a diagnostic, rather than therapeutic, role [218].

Another non-invasive strategy to enhance oocyte and early embryo competence is to support mitochondrial functions in in vitro cultured oocytes by supplementing the culture media with compounds upregulating the functions of histone deacetylases sirtuins. SIRT1 for instance stimulates mitochondrial activity, enhances its biosynthesis and regulates degradation of mitochondria [219].

### 4.4. IVF & ICSI

New evidence from genome-wide sequencing of neonatal cord blood has shown that epigenomes of ART newborns exhibit the loss of CpG methylation compared to those of natural conception (NC). A total of 176 genes were differentially methylated including genes employed in growth and neurodevelopment [195]. Similar findings were obtained from 7–9 week old fetal human tissue after elective termination of pregnancy. A total of 164 differentially methylated genes were detected and associated with the development of the skeletal system, body size, lipid, and steroid synthesis [220]. Moreover, a study on histone modifications between IVF and ICSI found significant differences in global H3K4me3. ICSI placentas had lower H3K4me3 levels than IVF placentas in line with its lower transcription activity [221]. It is important to bear in mind that ART vs NC comparison will always accompany epigenetic changes acquired during the in vitro embryo culture so no clear epigenetic input of IVF and ICSI can be drawn.

### 4.5. Embryo In Vitro Culture

In the female reproductive tract, particularly the oviducts, developing early embryos are under the influence of hormones, nutrients, growth factors, and cytokines [222]. The epigenome of in vitro cultured mammalian embryos is vulnerable to exposed culture conditions as aberrant DNA demethylation kinetics was detected in in vitro grown embryos compared to embryos of in vivo origin [183].

During in vitro culture, early embryos are exposed to limited nutrient availability and furthermore, they are influenced by the end products of their metabolism [223]. A stress response can be induced through the manipulation of embryos by pipetting, exposure to thermal stress, and/or detrimental change in pH [224,225]. It is evident that embryo in vitro culture conditions contribute to its epigenetic status. Embryo exposure to suboptimal culture conditions or toxic substances in the medium can result in altered DNA methylation, genetic reprogramming, developmental disruption, and consequently early embryo loss as demonstrated in the mouse, rat, and rabbit [183,226].

In vivo early embryonic development takes place in the oviducts, but in vitro embryo culture conditions are far from the oviductal environment, respective to nutrients, oxygen concentration, or epigenetic messengers. One example for both, oviductal extracellular vesicles (oEV) were shown to harbour miRNA that can downregulate specific mRNAs or modify gene expression is other ways [185]. These oviduct–embryo interactions are missing in vitro and would most likely play much more important roles in the regulation of the embryonic epigenome that await their discovery [227,228,229,230].

Adequate oxygen supply is another crucial condition for the success of in vitro culture. Concentrations of 5% oxygen and 20% oxygen are commonly used during in vitro culture of oocytes and early embryos. DNA methylation and gene transcription of mouse oocytes grown in in vitro conditions under 20% oxygen correlated more with the reality of in vivo conditions, indicating that higher oxygen concentration is beneficial for mouse oocytes matured in vitro [231]. In contrast, exposure of in vitro cultured bovine preimplantation embryos to 20% oxygen was associated with an increase in global DNA methylation indicating that in this case oxidative stress can alter the embryonic epigenome [178,179]. These findings suggest variations in the optimal oxygen concentration among different species. In order to optimize the oxygen in in vitro culture of mammalian embryos, it was suggested to reduce the concentration up to the physiological oxygen tension as the median oxygen rate in the mammalian oviduct is around 8% [232,233]. The importance of oxygen tension reduction during in vitro embryo culture was confirmed by systematic review and meta-analysis of published human ART studies, revealing an increase in pregnancy and live birth rates of embryos cultured at 5% oxygen concentration [234].

At 20% oxygen concentration in bovine embryo blastocyst culture, elevated ROS were detected [178]. Although the ROS are important signalling molecules in certain biological processes and are normal products of oocyte metabolism, they can interact with biological molecules such as lipids, proteins, and nucleic acids, cause oxidative stress and cellular damage leading to the impairment of oocyte quality [235]. Oxidative stress can also increase the risk of aberrant DNA methylation in in vitro cultured preimplantation embryos [236,237].

**Figure 5:**
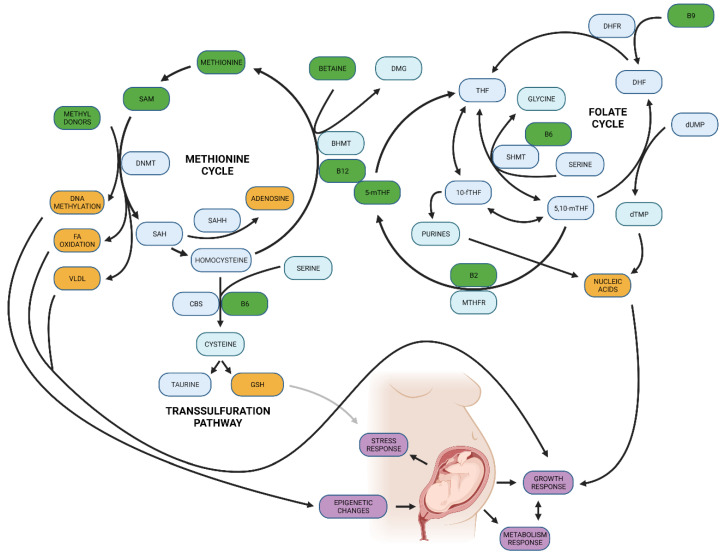
**One-carbon metabolism (OCM)-associated pathways in nutrition and epigenetics:** Many OCM compounds are nutritional supplements (green) such as B vitamins; Riboflavin (B2), Pyridoxine (B6), Folate (B9), or Cobalamin (B12). 5-methyl-tetrahydrofolate (5-mTHF) or methionine and betaine are another currently introduced nutritional supplements. OCM pathways produce molecules for epigenetic modifications [239], antioxidant production [240], and building compounds of nucleic acids, amino acids, or phospholipids (orange) [241], which are all together influencing in utero fetal development (purple). Abbreviations: 5,10-mTHF, 5,10-methenyl-tetrahydrofolate; 10-fTHF, 10-formyl-tetrahydrofolate; BHMT, betaine-homocysteine S-methyltransferase; CBS, cystathionine β-synthase; DHF, dihydrofolate; DHFR, dihydrofolate reductase; DMG, dimethylglycine; DNMTs, de novo and maintenance DNA methyltransferases; dTMP, deoxythymidine monophosphate; dUMP, deoxyuridine monophosphate; FA, fatty acids; GSH, reduced glutathione; MTHFR, 5,10-methylenetetrahydrofolate reductase; SAH, S-adenosylhomocysteine; SAHH, S-adenosylhomocysteine hydrolase; SAM, S-adenosylmethionine; SHMT, serine hydroxymethyltransferase; THF, tetrahydrofolate; VLDL, very-low density lipoprotein. The image was created with BioRender.com.

In order to compensate for the adverse conditions of in vitro culture and maintain DNA methylation, a supply of methyl donors is needed, e.g., folates, which are often absent in culture media [237]. Methionine, an important intermediate metabolite of the one-carbon metabolism pathway (OCM) (Figure 5), contributes to epigenetic regulation by providing methyl groups for DNA methylation via S-adenosyl methionine (SAM) [238]. The methionine level is an important factor influencing the quality of the early embryo epigenome. Elevated concentrations of the methionine product homocysteine in the oocyte and embryo environment is, however, harmful due to its toxicity and has to be converted back to methionine. Limited remethylation of homocysteine to methionine leads to a decrease of SAM causing DNA hypomethylation [238].

A high level of homocysteine was also detected in the serum of PCOS patients and in the follicular fluid of polycystic ovaries and was linked with poor oocyte quality [242,243]. The aging process may be an indirect factor contributing to the decrease of embryo quality and oocyte maturation through increasing homocysteine levels in follicular fluid; hence, a decrease of homocysteine level in follicular fluid can significantly improve the oocyte maturation rate and embryo quality [244]. Elevated homocysteine levels in follicular fluid is also associated with hypermethylation of mitochondrial DNA accompanied with a mitochondrial malfunction in oocytes retrieved from porcine polycystic ovaries [245]. In porcine in vitro cultured oocytes, exposure to homocysteine significantly reduced the survival rate, polar body extrusion rate, and cleavage rate; however, DNA methyltransferase inhibitor 5-AZA rescued the homocysteine-induced mitochondrial dysfunction and improved the quality of oocytes and developmental competence [245]

In vitro oxidative stress can also affect mitochondrial function. Treatment of in vitro cultured bovine oocytes with palmitic acid induced upregulation of peroxiredoxin 3, which is a mitochondrion-specific H_2_O_2_-scavenging enzyme, and elevation of the mitochondrial HADHB, UQCRB, and cytochrome C proteins suggesting that oxidative stress increased electron transport in bovine oocytes [180]. Exposure of MII mouse oocytes to H_2_O_2_ led to a decrease in mitochondria-derived ATP and disassembly of meiotic spindles [85].

### 4.6. Cryopreservation

Two main methods of germ cell cryopreservation exist: slow freezing and vitrification, both with the goal to eliminate ice crystal formation inside the cells. The slow freezing method is based on the continuous and steady decrease of temperature by 1–2 °C/min in the presence of cryoprotective agents and is conventionally applied for the preservation of human fertilized zygotes [246]. The second approach, called vitrification, is now the most widely used method for cryopreservation. It combines a rapid increase in media viscosity and solute concentration with snap freezing in liquid nitrogen. Vitrification has outperformed slow freezing in conventional ART human embryo cryopreservation by both higher clinical pregnancy and live births [247].

However, from the epigenetics perspective cryopreservation is still questionable [186]. Vitrifcation significantly reduced the ATP content in oocytes of various mammalian species, including humans [248,249]. Particularly changes in global DNA methylation, histone modifications and genetic imprinting are to be scrutinized. Research performed on vitrified mouse embryos found a common pattern of decreased global [250] and imprinted DNA [251] methylation. Vitrification of bovine oocytes was associated with a significant reduction in the expression profile of three epigenetic-related genes DNMT1, DNMT3B, and HDAC1 [252]. In vitrified porcine embryos, a greatly reduced expression of epigenetically associated key genes SMYD3, TET2, and HDAC8 led to altered epigenetic reprogramming and decreased blastocyst rates [253]. The cryoprotective agents present in IVF vitrification media might negatively affect the epigenetic profile in embryos, as dimethylsulfoxide (DMSO) was found to be responsible for the disruption of global DNA methylation and significant decrease of ATP content in vitrified human cardiac tissue [254]. A detailed analysis of molecular changes occurring in cryopreserved germ cells and embryos is necessary to distinguish possible molecular targets that could contribute to improve the cryopreservation procedures.

## 5. In Utero Epigenetics, beyond ART?

Exposure to unfavorable conditions before pregnancy and during intrauterine development lead inevitably to epigenetic alterations of a newborn and can evolve into pathogenesis of metabolic, cardiovascular, endocrine, and malignant disorders in adulthood [255]. As every ART-produced embryo has to be eventually planted into a mother’s womb, these in vivo epigenetic factors deserve a closer look in order to understand the full story.

### 5.1. Endometrial Receptivity & Placentation

Following ART embryo transfer, each embryo faces the selective process of nidation and implantation into the endometrial tissue of the uterine wall. This in utero process itself is highly complex and involves the cooperation of many signalling pathways. Many epigenetical mechanisms are involved as well, such as DNA methylation [256], m^6^A methylation [166], or interaction with ncRNAs secreted from exosomes [257]. It has even been suggested that DNA methylation profiles of cervical secretion could serve in the future as an alternative way for diagnosing endometrial receptivity [258]. Decidualization, the process of endometrial preparation for blastocyst implantation, is suppressed in human endometrial stromal cells by an ncRNA, the miR-542-3p. Overexpression of this miRNA also downregulates VEGF, cyclooxygenase-2 (COX-2), and matrix metalloproteinase (MMP-9), all linked with angiogenesis [259]. Most ncRNAs are able to exert their effects by transportation as cargos in lipophilic exosomes. In this way, ncRNAs influence the embryonic development in the oviduct and uterus [257]. So far, little is known about the scope of the influence of these ncRNAs on the epigenome of a developing individual.

Following successful implantation, the embryo turns to cardiac and neural development. These processes are energy-dependent and highly susceptible to proper dietary intake and environmental conditions. Defective placentation is associated with impaired mitochondrial function and associated low ATP production [82,83]. Incorrect DNA methylation on imprinted genomic regions dysregulates placental function [99,100]. Nutritional and environmental status can influence the uterine and fetal epigenome to a large extent, and therefore their effects have to be taken seriously into account.

### 5.2. Nutritional Epigenetics

Numerous studies describe the effects of nutrition on the epigenome during embryonic development (reviewed in [238,260,261]). Particularly, the nutritional source of dietary methyl donors in early development can influence the DNA methylation process [239]. Methyl groups or so called one-carbon groups are produced through OCM, which integrates folate and methionine cycles and is a source for epigenetic DNA methylations, biosynthesis of DNA, proteins, and lipids. Amino acids such as methionine, glycine, and serine and appropriate levels of especially B class vitamins (B2, B6, B12) and folic acid (B9) are integral inputs for the successful functioning of one-carbon metabolism (Figure 4). Epigenetic changes in humans associated with OCM modifications affect pathways related to growth, metabolic functions, neural development, and stress response [239].

The modulation of epigenetic modifications, including DNA methylation, is done by the mTOR signalling through the OCM [262]. mTOR signalling is able to affect OCM by increasing the de novo synthesis of serine, one of the major single carbon donors [263]. Elevation of placental mTOR observed in obese women increased birth weight [264]. As one of the main functions of OCM is to produce S-adenosylmethionine (SAM) to ensure methyl-group transfer reactions, mTOR signalling, by influencing the OCM, is able to influence epigenetic modifications, including DNA methylation [262]. The epigenetic regulator methyltransferase DNMT1 is one of the downstream targets of the mTOR pathway [265]. The experimental inhibition of mTOR induced the suppression of DNA methyltransferase DNMT1 [260].

Microelements in the diet, e.g., Cu, Mn, Se, and Zn are often required for proper enzymatic function, neutralizing ROS, and are also involved in epigenetic regulation. For example, zinc is implicated in the correct functioning of methyltransferases and methyl-binding proteins and its deficiency has been suggested to affect the activities of zinc-dependent epigenetic enzymes, which are essential for DNA methylation [261]. Prolonged dietary Se supplementation in rats affected global and specific DNA methylation in liver and colon tissue [266]. Mn tends to accumulate in the placenta, and its supplementation experiments in chick embryos counteracted hyperthermic stress effects by modulating DNA methylation and histone acetylation [267]. A recent US study found a similar mode of action in Cu metabolism, which may be employed in DNA methylation and the regulation of human placentation [268].

Malnutrition can epigenetically induce in utero obesity in offspring, which usually manifests in adulthood. Blood analysis of human adults revealed that periconceptional exposure to famine altered the DNA methylation of genes implicated in growth and metabolic regulation. Prenatal famine exposure resulted in changes of DNA methylation patterns in genes associated with cell growth, metabolic health, mitochondrial function, adipogenesis, and its deposition [269,270]. A preovulatory protein restriction diet in rats induced abnormal mitochondrial ultrastructure in oocytes and negatively affected gene expression related to mitochondrial biogenesis [271].

This demonstrates epigenome vulnerability by famine in the early stages of development. There is strong evidence that maternal nutrition influences the development and future health of offspring. Studies done on mice [272] and cattle [273] confirmed maternal diet effects on oocyte DNA methylation. Postpartum cows exposed to negative energy balance and metabolic stress had a number of maternally imprinted genes in their oocytes hypomethylated [274].

Maternal obesity is another risk factor affecting offspring health epigenetically and has been shown when together with excessive nutrition intake to have a positive correlation with offspring obesity [275]. Causes of fetal overgrowth have been explored on the mouse model. It was documented that obesity in pregnancy is linked to stimulation of placental insulin/IGF-1/mTOR and leptin signalling pathways [163]. The obesity mouse model resembles the changes in placental mTOR signalling and amino acid transporters activity observed in obese women giving birth to large babies [276]. DNA hypermethylation in the placentas of obese pregnant women was associated with reduced expression of the ten-eleven translocation (TET) methylcytosine dioxygenases, enzymes involved in DNA demethylation [58]. Increased gestational weight in early pregnancy is related to the enhanced CpG methylation of *MMP7, KCNK4, TRPM5,* and *NFKB1* genes in offspring cord blood DNA [277].

Maternal obesity and overnutrition affect mitochondria function and induce epigenetic changes of mtDNA. The mtDNA copy number, elevated expression of nuclear genes encoding mtDNA transcription factors *Tfam and Nrf1* were detected in oocytes of obese mice [278]. A maternal obesogenic diet (high fat/high sugar) was associated with elevated mtDNA content and increased expression of mtDNA biogenesis regulators *Tfam* and *Pgc-1α*, enhanced mitochondrial antioxidant defence, increased lipoxygenase expression, enhanced expression of transcriptional regulator NF-κB, and depletion of ovarian follicular reserve in young adult female mouse offspring [279]. The analysis of the newborn umbilical cord indicated that promoter methylation of the mitochondrial biogenesis regulator *PPARGC1A* in babies was positively correlated with maternal BMI [280]. It is evident that maternal obesity may affect the offspring metabolism through epigenetic regulation of specific genes.

It is common knowledge that consuming alcohol in pregnancy affects embryo development and can induce a variety of birth defects and neuronal disorders in offspring [281]. Alcohol intake interferes with normal folate metabolism (Figure 5) and decreases folate bioavailability for methyl donors by inhibiting methionine synthase and methionine adenosyl transferase [282].

It has been shown that alcohol metabolites, such as acetaldehyde, modify DNA methylation by inhibiting DNA methyltransferases [283].

The effect of alcohol abuse on the methylation of specific genes resulted in alterations of gene expression and neural development as reported in numerous studies (reviewed in [284]). In in vitro cultured fetal mouse neurons, the alcohol exposure induced a decrease of DNA methylation detected in the vicinity of the NMDA receptor subunit NR2B gene, which plays an important role in neural development and in learning and memory [285,286].

In human oocytes, alcohol-associated epigenetic changes were detected already in the growth phase when genomic imprints are established and could possibly affect the health of the child [287].The in vivo exposure of mice embryos to ethanol resulted in retardation of embryo development and was accompanied by epigenetic alteration of the *H19/Igf2* methylation in the placenta; the paternal allele of *H19/Igf2* was less methylated while the methylation of the maternal allele was elevated [288]. In mouse embryos exposed to alcohol, in vitro changes in methylation on chromosomes 7, 10, and X related to neural tube defects were detected [289].

## 6. Fetal Epigenetics Dependence on Maternal Lifestyle and Environment

In the present day, people live in a highly stressful world in compromised living environments. As we have already mentioned, both undernutrition and overnutrition can impact one’s epigenome irrespective to ART. Here, we emphasize that postponing parenthood or living in physically and psychologically toxic environments can induce changes to the new generation’s epigenome (Table 2).

### 6.1. Lifestyle

Advanced maternal age negatively influences oocyte maturation, meiotic divisions, and embryonic development [306,307]. Increasing maternal age raises the chances of miscarriage and adverse health issues in offspring, mainly due to chromosomal aneuploidies such as Down’s syndrome [308]. Age-related decrease of ooplasm quality, mitochondrial defects, and abnormalities in meiotic maturation mechanisms are possible causes of the advanced maternal age-related decline of oocyte competence [168,309,310] A higher incidence of chromosomal abnormalities was reported in mammalian oocytes acquired from aged females (reviewed in [311]), and an elevated occurrence of aneuploidy related diseases was observed in babies born to mothers over 35 years of age [309]. The meiosis-specific cohesin subunits, REC8 and SMC1B, were found to be decreased in oocytes of women aged 40 and over, suggesting that age-related decrease of meiotic cohesin subunits impair sister chromatid cohesion and results in increased segregation errors [295]. The effect of maternal age on oocyte quality and associated epigenetic changes have been well-documented and extensively reviewed in humans [310].

Oocytes of older women (41–44 years old) were more prone to oxidative damage by the attenuated expression of cytochrome c oxidases (COX gene family) involved in oxidative phosphorylation and energy production and down-regulation of members of the peroxiredoxin gene family [296]. Similarly, gene expression analysis of ovaries in aged mice revealed a decrease in mRNA expression of mitochondrial antioxidant genes, peroxiredoxin 3 (Prdx3), and thioredoxin 2 (Txn2) [92]. Gene transcriptome analysis of human oocytes retrieved from patients older than 40 years revealed a decreased expression of spindle checkpoint genes, DNA damage checkpoint-related genes, ADP ribosylation factors involved in protein trafficking, and mRNA coding for the EIF4ENIF1 protein, which mediates the nuclear import of eIF4E [294]. Closer analysis of MII aged mouse oocytes gene expression (42–45 weeks old) revealed a downregulation of genes involved in mitochondrial functions, antiapoptotic mechanisms, and those involved in the ubiquitin-proteasome degradation pathway. Moreover, expression was reduced in transcripts related to microtubule cytoskeleton, chromosome segregation, and maintenance of the DNA methyltransferases *Dnmt1o* and *Dnmt1s* [298]. Hence, reduced DNA methylation in MII oocytes and early embryos of aged mice together with a low abundance of DNA methyltransferases clearly points to a lower reproductive potential [297,312]. More recently, it was suggested that decreased DNA methylation related to advanced maternal age may partially induce significant changes to gene expression and alter developmental fitness (reviewed in [167,313]).

Maternal smoking in pregnancy remains a serious issue that gravely affects child health. Fetal exposure to maternal smoking during pregnancy induces changes in DNA methylation of different tissues. The impact of prenatal exposure to tobacco smoke on DNA methylation was mostly analyzed in the cord blood and placenta of newborns—reviewed in [314]. It was found that DNA methylation patterns associated with smoking are relayed to a low birthweight [315] and schizophrenia induction in adulthood [316]. Moreover, the meta-analysis mapping association between maternal smoking in pregnancy and newborn blood DNA methylation revealed that smoking whilst pregnant causes changes in the CpGs methylation of numerous genes including those involved in teeth and neurologic development as well as cancer induction [293]. *AHRR* and *CYP1A1,* genes of aryl hydrocarbon receptor signalling, which is engaged in detoxification, were also found to be differentially methylated in the cord blood of newborns exposed to maternal smoking [317].

Lifestyle can also be a factor determining the occurrence of gestational diabetes mellitus (GDM) in women. GDM is associated with an increased risk of cardiometabolic diseases and diabetes in the offspring [301]. DNA collected from venous blood of GDM women offspring detected differentially methylated CpGs in genes associated with type 2 diabetes, diabetic nephropathy, obesity, and coronary heart disease [301]. It has been shown that pregestational hyperglycemia renders the offspring more vulnerable to glucose intolerance. The expression of TET3 dioxygenase, responsible for 5-methylcytosine oxidation and DNA demethylation in the zygote, is decreased in oocytes from a mouse model of hyperglycaemia (HG mice) and in people with diabetes [318].

### 6.2. Environment

Endocrine Disrupting Chemicals (EDCs) cause serious defects in human health. EDCs are chemicals of natural or man-made origin that interfere with the endocrine system. Humans are exposed to EDCs from many sources, including diet, thermal receipt papers, cosmetics, cleaning products, pesticides etc. [319]. Exposure to EDCs during development can induce permanent alterations of physiology and increase predisposition to health issues such as obesity, asthma, and cancer [320].

Bisphenol A (BPA) is a ubiquitous plasticizer, EDC with probable estrogen-like activity. Newborn cord blood DNA studies revealed that prenatal exposure to BPA (but not bisphenol F and bisphenol S) induces hypomethylation of gene promoters related to adipogenesis, growth and metabolism [321]. Hypomethylation of the obesity-associated mesoderm-specific-transcript (*MEST*) gene promoter enhanced *MEST* expression, which resulted in a significant increase of body mass index (BMI) in children [303]. Exposure to BPA during the late stages of oocyte development and the early stages of embryonic development disrupted the expression of imprinted genes in mouse embryos and placentas [304]. Mouse BPA exposure at 9–16 day of pregnancy led to decreased methylation and enhanced expression of the homeobox gene *Hoxa10* in offspring, a key regulator of in utero organ development [322]. Additionally, polystyrene nanoparticles inhibit meiotic maturation by negatively affecting spindle assembly and chromosome alignment in mice oocytes; moreover, exposure to polystyrene nanoparticles increased oxidative stress and mitochondrial aggregation during meiotic maturation [305]

Stress is a widespread environmental factor affecting human reproduction. Preconception and pregnancy time exposed to stress is associated with developmental problems and new-born physical and psychological health. Maternal stress has been linked to infant mortality, premature weight and low birth weight [323]. Increased DNA methylation of the glucocorticoid receptor NR3C1 gene promoter, which is related to maternal stress via controlling hypothalamic-pituitary-adrenal axis (HPA) has been reported in human cord and new-born blood [324]. Chronic maternal distress in pregnancy was accompanied by altered CpG methylation on glucocorticoid pathway genes in human placentas, which suggests that the placenta can be the main mediator between maternal and fetal stress. [299]. Similarly, differential DNA methylation of the HPA axis genes *CRH* and *NR3C1* was detected in cord blood of new-borns and *CRH, CRHBP, NR3C1*, and *FKBP5* in their placentas [325].

## 7. Conclusions

Over the past years, an increasing research interest has been focussed on understanding the regulation of the animal and human epigenomes. Many publications have demonstrated that DNA and RNA methylation, histone modifications, and non-coding RNA regulation are integral to the normal embryo development and future health of a newborn. However, our current understanding of these mechanisms is unsatisfactory. Here, we endeavoured to emphasize the connection between assisted reproduction technology and its epigenetic implications for oocyte and embryo development. We did not want to overlook the fact that following an embryo transfer, the in utero development is epigenetically affected by the actual fitness and age of the reproductive system as well as by external stimuli such as diet or nutrition. We want to show a complex picture of what is behind ART live-birth rates and the resulting health implications from the epigenetic point of view.

ART is of enormous importance to infertile couples and our society in general. Therefore, it is much needed to focus on improving ART and minimizing unnecessary negative impacts by discussing and bringing in the latest knowledge of epigenetic mechanisms involved in clinical infertility treatments. Many of the questions regarding epigenetics influencing in vitro oocyte and embryo culture protocols remain unanswered. Future studies should search for epigenetic key points in the concerned developmental pathways and investigate their clinical relevance as biomarkers or new treatments.

## Figures and Tables

**Table 1:** Summary of effects induced by ART on epigenetic changes in oocytes, early embryos and their influence on offspring. “NE” indicates specific gene effects were not evaluated.

Stressor	Species	Genes Affected	Main Findings	Reference
		**Ovarian Stimulation**		
Controlled ovarian stimulation	human	NE	Chromosomal aneuploidy	[168]
Superovulation	mouse	*Dnmt1, Dnmt3A, Dnmt3B*	Affected expressin of methyltransferases in GV, MII oocytes, in one-cell and two-cell embryos	[169]
Superovulation	mouse	*Epab, Pabc1*	Altered expression of translational regulators *mRNA* in mouse GV and MII oocytes and in zygots	[170]
Superovulation	mouse	*Snrpn, Peg3, Kcnq1ot1, H19*	Disrupted methylation of imprinted genes in blastocysts	[171]
Superovulation	mouse	*Gfod2, Foxi3, Celf4, Syf2*	In oocytes, altered methylation of genes involved in glucose metabolism, nervous system development, cell cycle, cell proliferation, and mRNA processing	[172]
Superovulation	mouse	*H19*	Altered *H19* methylation in mouse blastocysts after in vivo fertilization	[173]
Superovulation	mouse	*Fasn, Dgat1, Dgat2*	Decreased fatty acid content in mice 2-cell embryos by reducing the *Fasn* and increasing the *Dgat1* and *Dgat2 expression*.	[174]
Repeated superovulation	mouse	*Cox1, Cytb, Nd2, Nd4*	Altered expression of mitochondrial genes in mouse cumulus cells	[175]
Repeated superovulation	mouse	NE	Abnormalities in mitochondrial structure and distribution in mouse oocytes	[176]
Superovulation	mouse	NE	Decrease of mitochondrial activity and ATP production in mouse oocytes	[177]
Superovulation	bovine	*TXN2, PDX3*	Decline of mtDNA copy number in bovine oocytes., decreased expression of antioxidant genes in bovine cumulus cells	[93]
		**Oxidative stress**		
Presence of reactive oxygen species	human	NE	Sperm originated changes to epigenetic regulation of human embryo development	[178]
Culture under 20% of oxygen	bovine	*CAT, GLRX2, HSP90AA1. KEAP1, NFR2, PRDX1, PRDX3, SOD1, TXN, TXNRD1, H2AFZ, H3F3B*	Increase of transcript of genes associated with epigenetic remodelling, oxidative stress and cellular stress response in blastocysts	[179]
Culture under 20% of oxygen	bovine	*DNMT3A*	Elevated DNMT3A expresiion and increase of global DNA methylation in 4-cell embryos and blastocysts	[180]
Oxidative stress (palmitic acid)	bovine	*PRDX3, HADHB, UQCRB, CYCS*	Upregulation of PRDX3 protein. Elevation of the mitochondrial HADHB, UQCRB and CYCS proteins in oocytes	[181]
Oxidative stress (H_2_O_2_)	mouse	NE	Decrease in mitochondria-derived ATP and disassembly of spindles in in vitro cultured MII oocytes	[85]
		**In vitro techniques**		
Oocyte in vitro maturation	human	*HDAC1*	Compromised deacetylation in oocytes. Residual acetylation linked to aneuploidy	[182]
Oocyte in vitro maturation	bovine	*SIRT2*	Faulty mitochondria	[183]
Cytoplasmic transfer	human	Not tested yet	10–15% cytoplasm transfer into aged oocytes prroduced healthy offspring	[95]
Suboptimal culture media	rabbit	NE	Alteration of DNA methylation reprogramming in paternal pronuclei of zygotes	[184]
In vitro fertilization & ICSI	human	H19	ART caused demethylation resulted in the changes of genomic imprinting	[185]
Embryo in vitro culture	human	NE	miRNAS detected in spent culture medium downregulate embryonic mRNAs	[186]
Cryopreservation	human	*LINE1*	Differently methylated placental DNA between fresh and frozen embryotransfers	[187]
Suboptimal culture media	mouse	NE	Higher methylation disturbances in embryos from superovulated females and IVF	[188]
Intracytoplasmic sperm injection	mouse	*H19, Snrpn, Peg3, Igf2*	Imprinting defects in somatic tissues	[189]

**Table 2:** Summary of lifestyle, diet, and environment effect on epigenetic changes in oocytes, early embryos, and their impact on offspring. “NE” indicates specific gene effects were not evaluated.

Stressor	Species	Genes Affected	Main Findings	Reference
	**Undernutrition**			
Periconceptional exposure to famine	human	*IGF2*	*IGF2* hypomethylation in adults 60 years later	[290]
Periconceptional exposure to famine	human	*IL10, INSIGF, LEP, MEG3, ABCA1, GNASAS*	Altered DNA methylation of genes implicated in growth and metabolic regulation	[270]
Prenatal exposure to famine	human	*ABCG1, PFKFB3, METTL8*	Altered DNA methylation of genes associated with lipid metabolism, glycolysis, and adipogenesis in adults	[269]
Low levels of dietary methyl donors during embryonic development	human	NE	Affected DNA methylation process and impact on postnatal long-term health	[291]
Protein restriction during pregnancy	mouse	*Lep*	Increased *Lep* promoter methylation and decreased leptin expression in offspring	[292]
Preovulatory protein restriction	rat	*Drp1, Opa1,* *Mfn1/2, Parl, Ndufb6, Hk2*	Altered expression of genes involved in mitochondrial biogenesis in superovulated oocytes	[271]
Negative energy balance and metabolic stress	bovine	NE	Hypomethylation of maternally inherited imprinted genes in oocytes of postpartum cows	[274]
	**Obesity**			
Obesity in pregnancy	human	*TET1, TET2. TET3*	DNA hypermethylation and reduced expression of methylcytosine dioxygenases in placenta	[58]
Gestational weight gain	human	*MMP7,KCNK4,TRPM5* and *NFKB1*	Increased DNA methylation in offspring.	[277]
Obesity in pregnancy	mouse	*IGF-1, mTOR, LEP*	Stimulation of placental insulin/IGF-1/mTOR and leptin signalling pathways	[163]
	**Alcohol, smoking**			
Alcohol	mouse, human	NE	Birth defects and neuronal disorders in offspring	[281,284]
Alcohol	mouse	*Cyp4f13*, *Nlgn3, Elavl2, Sox21, Sim1, Igf2r, Hist1h3d*	Decreased methylation of genes associated with development, imprinting and chromatin in embryos exposed to ethanol in vitro	[289]
Alcohol	mouse	*H19/Igf2*	Retardation of embryo development in vivo and alteration of the *H19/Igf2* methylation in placenta	[288]
Maternal smoking	human	*BMP4, BMHT2, DLGAP2, PRDM8, NRP2, ESR1, IL32, HOXB2*	In newborns, changes in CpGs methylation of genes involved in tooth and neuronal development and in cancer induction	[293]
	**Maternal age**			
Advanced maternal age (more than 40 years)	human	*BUB1B, BUB3, MAD3, BUB1, REC8, ATR, CHEK1, NBS1, RAD17, EIF4ENIF1*	In oocytes, reduced expression of spindle checkpoint and DNA damage checkpoint-related genes, lowered mRNA expression of the nuclear import mediator of eIF4E	[294]
Advanced maternal age (more than 40 years)	human	*REC8, SMC1B*	Decreased expression of the meiosis-specific cohesins components, REC8 proteins, and SMC1B in oocytes	[295]
Advanced maternal age (41–44 years)	human	*PRDX1, PRDX2, PRDX4, PRDX6* *COX5A, COX7B, COX8A, COX8C, COX11, COX14, COX17*	Down-regulation of the peroxiredoxin gene family members and attenuated expression of the cytochrome c oxidases in oocytes	[296]
Advanced age	mouse	*Dnmt3a, Dnmt3b, Tfam*	Downregulation of maintenance DNA methyltransferases and mitochondrial transcription factor in oocytes	[297]
Advanced age	mouse	*Hook1*, *Tuba1*, *Tubd1*, *Dncic2*, *Kif3*, *Rnf19*/*Dorfin*, *Pcnt2*, *Nin, Smc4l1, Dnmt1o, Dmap1, Dnmt3L*	Decrease of transcripts related to microtubule cytoskeleton and chromosome segregation, downregulation of methyltransferases in oocytes	[298]
	**Other causes**			
Maternal stress during pregnancy	human	*HSD11B2*, *NR3C1, FKBP5*	Increased methylation and expression of glucocorticoid pathway-related genes in placenta and children blood.	[299,300]
Maternal gestational diabetes	human	*PDE6A*, *PRKCZ*, *PVT1*, *GALNT2*, *MS4A3*, *IL1RN*, *BTD*	In children, differentially methylated genes associated with type 2 diabetes, obesity, diabetic nephropathy, and coronary heart disease.	[301]
Eleveted homocysteine level	porcine	*mtDNA (12S, 16S rRNA and ND4)* *ND1, ND4L, ND5, COX1, CYTB mRNA*	Hypermethylation of mtDNA in oocytes from PCOS ovaries	[302]
	**Pollutants**			
Bisphenol A	human	*MEST*	Hypomethylation of the obesity-associated mesoderm-specific-transcript (*MEST*) gene promoter and enhanced *MEST* expression in children	[303]
Bisphenol A	mouse	*Snrpn, Ube3a, Igf2, Kcnq1ot1, Cdkn1c, Ascl2*	Disruption of imprinted gene expression in embryos and placentas.	[304]
Polystyrene	mouse	NE	Negative effect on oocyte spindle assembly and chromosome alignment, increased oxidative stress, and mitochondrial agregation	[305]

## Data Availability

Not Applicable.
